# Reconstruction of Gustilo type IIIC tibial open fracture caused by shotgun injury using combination Masquelet technique and cross-leg pedicle flap

**DOI:** 10.1016/j.ijscr.2020.06.036

**Published:** 2020-06-13

**Authors:** Chun-Yen Chen, Yung-Cheng Chiu, Cheng-En Hsu

**Affiliations:** a Department of Orthopedic Surgery, China Medical University Hospital, Taichung, 40447, Taiwan; b School of Medicine, China Medical University, Taichung, 40447, Taiwan; c Department of Orthopedics, Taichung Veterans General Hospital, Taichung, Taiwan; d Sports Recreation and Health Management Continuing Studies-Bachelor’s Degree Completion Program, Tunghai University, Taichung, 407, Taiwan

**Keywords:** Cross-leg pedicle flap, Masquelet technique, Shotgun injury, Open proximal tibial fracture

## Abstract

**Introduction:**

The shotgun injury may cause large-scale bone and soft tissue destruction especially when people get shot from a very close range. Here we present a case of Gustilo type IIIC open fractures of the proximal tibia with vascular injury, large bone and soft tissue defect treated with Masquelet technique in combination with cross-leg pedicle flap.

**Presentation of case:**

A 34-year-old man presented with open proximal tibial fracture of Gustilo type IIIC, AO/OTA (Arbeitsgemeinschaft für Osteosynthesefragen/Orthopaedic Trauma Association) classification 41-C2.3, caused by a shotgun injury from very close range (2 m). The wound was complicated with anterior tibial artery injury and segmental bone loss. After radical debridement, the fracture was temporarily stabilized with external fixator, followed by double plate fixation with cement spacer filled into the bone defect in accordance with the Masquelet technique. The soft tissue defect was covered with a cross-leg pedicle flap. At 3 months after trauma, the cement spacer was replaced by mixed autologous and synthetic bone graft. The fracture had successfully healed by 12 months after trauma.

**Discussion:**

When the integrity of lower leg vessels is doubtful, the Masquelet technique in combination with cross-leg pedicle flap is an effective and safe alternative treatment for this condition.

**Conclusion:**

We recommend to manage Gustilo type IIIC open fractures of the proximal tibia with bone defect with Masquelet technique in combination with cross-leg pedicle flap especially when the free flap technique is relatively contraindicated or trauma surgeon is not a highly-skilled microsurgeon.

## Highlights

• The treatment of open fracture combined with artery injury is challenging.
• In case a free flap is contraindicated, the cross-leg flap is a good optein.
• Masquelet technique is a promising treatment for bone defect with artery injury.

## 1 Introduction

The shotgun injury may cause large-scale bone and soft tissue destruction especially when people get shot from a very close range [1,2]. Traditionally, open fracture with large bone and soft tissue defect can be treated via serial debridement, external fixation and vascularized bone graft. However, when vascularized bone graft is not available or contraindicated, alternative treatment option is necessary.

Very few studies have reported clinical and radiographic outcomes of Gustilo type IIIC open fractures caused by shotgun injury [3]. Here we present a case of Gustilo type IIIC open fractures of the proximal tibia with large bone and soft tissue defect treated with Masquelet technique in combination with cross-leg pedicle flap.

## 2 Case report

The case report has been written according to the SCARE 2018 guidelines [4].

**Fig. 1:**
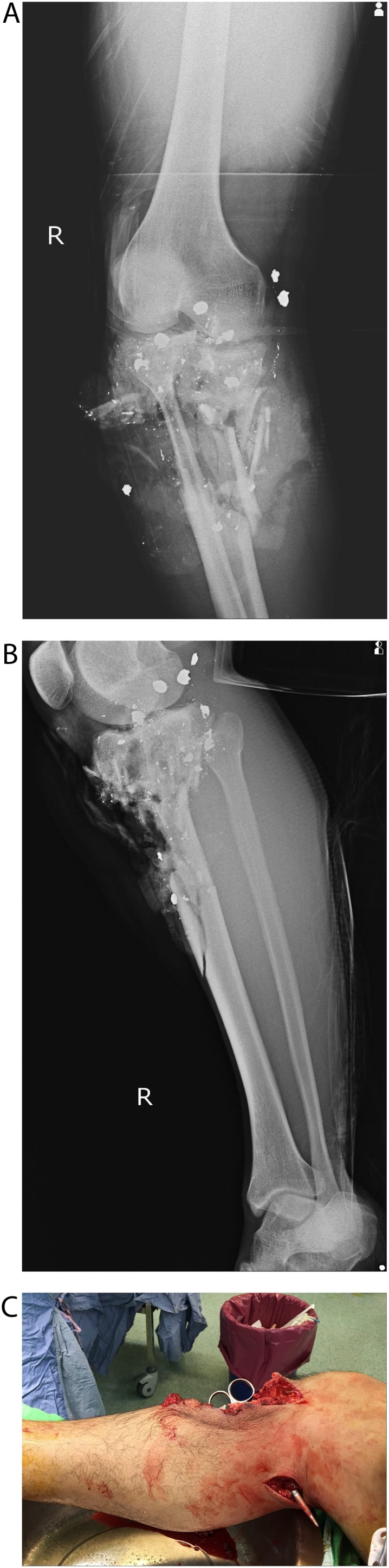
(A) Preoperative radiograph, AP view (B) Lateral view (C) Initial soft tissue loss caused by gunshot injury. The direction of the scissors indicates the bullet trajectory.

**Fig. 2:**
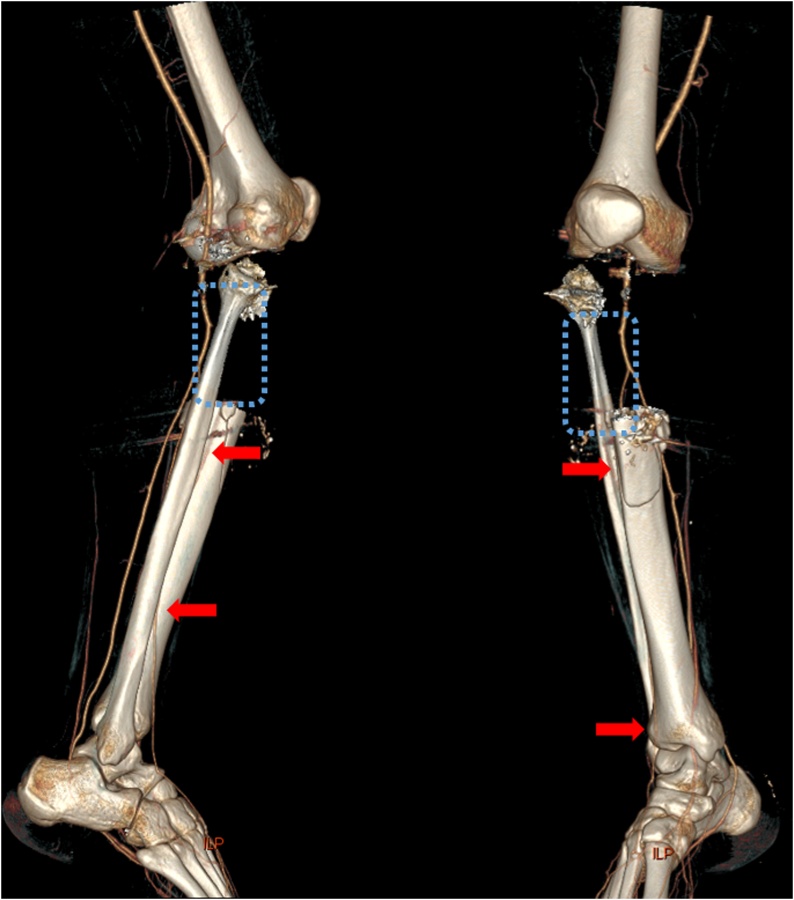
Computed tomography angiography at right knee with the reduction of metallic artifact. (Red arrow: anterior tibia artery with contrast enhancement, Blue dash box: missing segment of anterior tibia artery with poor contrast enhancement).

A 34-year-old man suffered a shotgun attack from 2 m away on his right proximal tibia. Clinical examination revealed a Gustilo type IIIC open fracture. The pellets entered from the posterior calf and came out the anterior side of the tibia, creating a 15 × 20 cm^2^ open wound. Radiographs indicated comminuted fracture with bone loss about 7 cm and multiple shotgun pellets (Fig. 1). Computed tomography angiography revealed poor contrast enhancement in the right anterior tibial artery (Fig. 2).

**Fig. 3:**
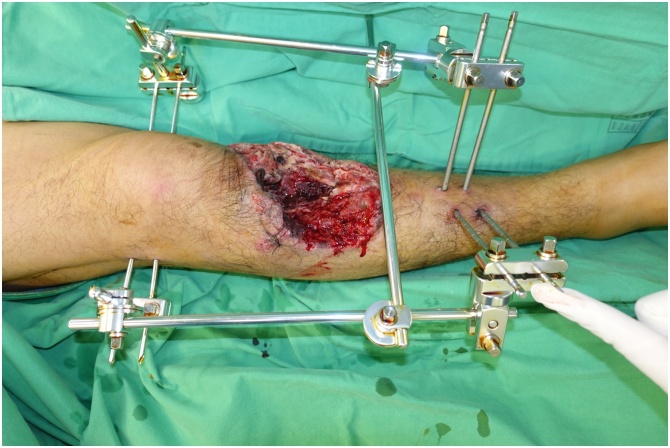
Large soft tissue defect (20 cm × 15 cm) of the anterior border of the tibia after primary wound debridement.

Initial wound debridement with fixation by external fixator were done 2 h after the trauma (Fig. 3). The shrapnel fragments and dead bone were removed thoroughly. Subsequent wound exploration revealed segmental loss of anterior tibial artery. On day 3, day 6, day 9 and 14 after trauma, four times of debridement were performed sequentially and clean and well-perfused wound bed was noted.

**Fig. 4:**
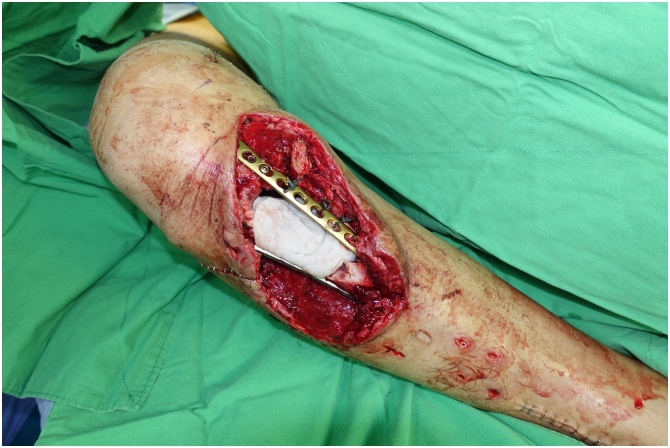
Antibiotic-loaded PMMA spacer used to fill the bone defect and double locking plates used to fix the fracture.

**Fig. 5:**
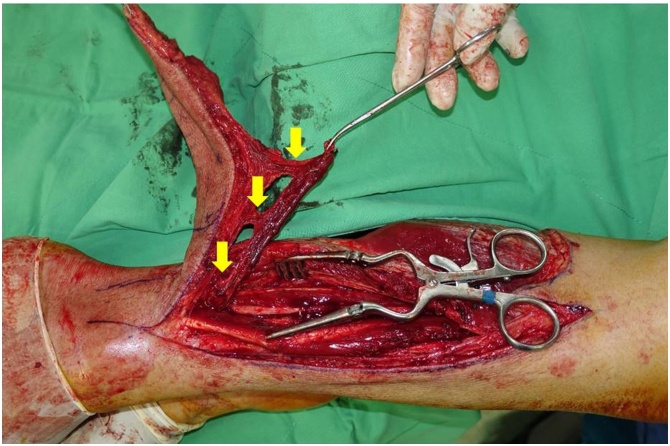
Cross-leg pedicle flap nourished by posterior tibial artery and three perforator vessels marked with yellow arrow signs.

**Fig. 6:**
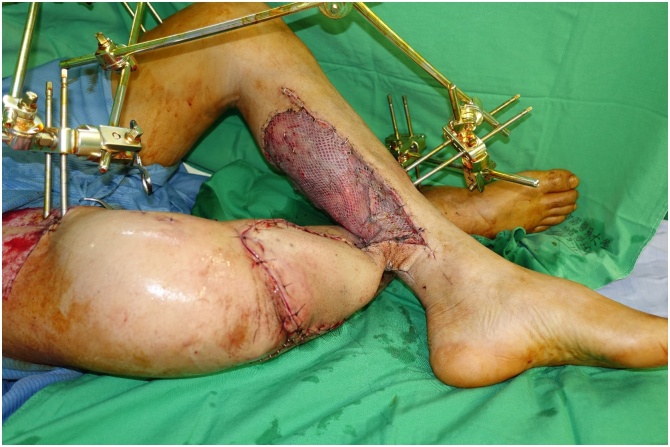
Donor site covered with split thickness skin graft.

On post-trauma day 16, we fixed the tibial fracture with two locking plates (lateral: NCB Proximal Tibia System, Zimmer Biomet; medial: 3.5-mm Locking Compression Plate, DePuy Synthes). We filled the bone defect with vancomycin and gentamicin loaded cement spacer (Fig. 4). The skin defect was covered with a cross-leg pedicle flap. The flap is nourished by the posterior tibial artery and three perforator vessels (Fig. 5). We immobilized the two legs with cross-leg external fixators (Fig. 6). The pedicle of the cross-leg flap was divided and the cross-leg external fixator was removed 3 weeks after the flap transplantation. Partial weight bearing was allowed after the flap dividing surgery.

**Fig. 7:**
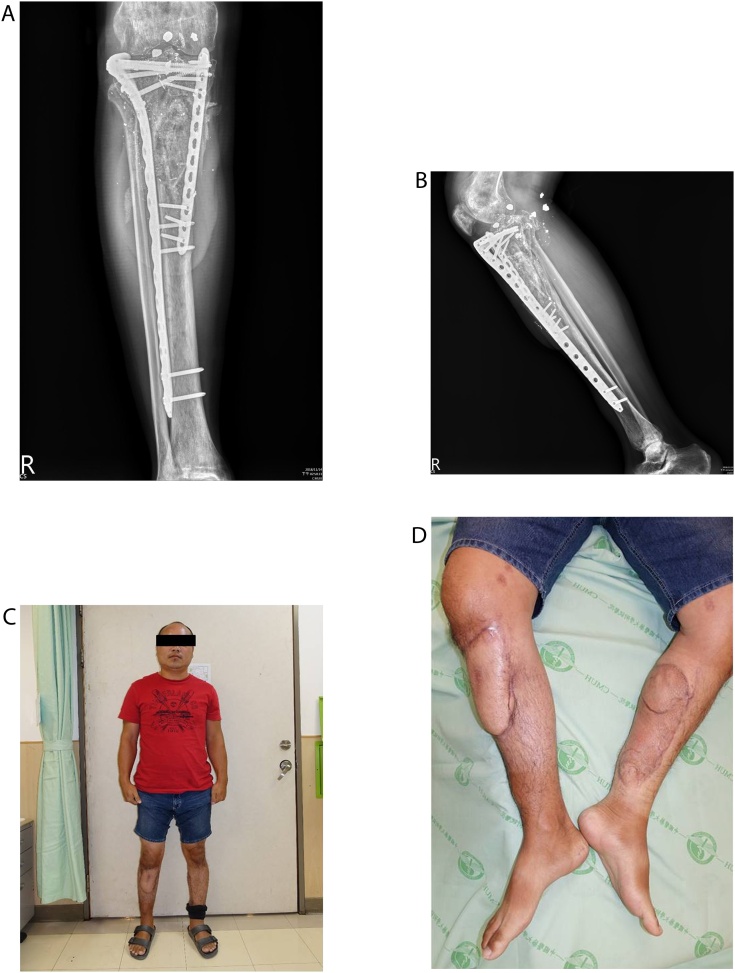
(A) Radiograph at 12-months-follow-up, AP view and (B) lateral view (C) Patient is able to stand without leg length discrepancy (D) Well-healed skin graft and flap on both lower extremities.

We replaced the cement spacer with mixed autogenous and synthetic bone graft at 12 weeks after trauma [5,6]. At 12 months, bone healing was observed on the radiograph (Fig. 7). The range of motion of right knee was achieved to 0–120 degree. The patient referred no pain during the daily activities.

## 3 Discussion

The treatment of open fracture involves segmental bone loss and large soft tissue defect along with major vessel injury caused by shotgun injury is challenging. Free vascularized osteocutaneous flap such as fibula and iliac crest flap have been reported as reliable flap with satisfactory results [7]. However, the integrity of lower leg vessels is mandatory for the success of this treatment. The injured anterior tibial artery with large trauma zone was an obvious contraindication for free flap reconstruction.

Bone transport is also an effective technique for long bone defects. However, the prolonged external fixator use may cause stiffness of ankle and knee, low quality of life and potential pin tract infection are also the drawbacks.

Generally, nonvascularized bone graft is not advocated for reconstruction in bone defects over 5 cm. Masquelet et al. introduced a technique to treat bone defects of more than 5 cm with non-vascularised bone graft in 2000 [5]. This technique comprises two stages of operation. The first stage of the Masquelet technique includes radical debridement, soft tissue repair, and implantation of a cement spacer into the bone defect. The second stage is performed 6–8 weeks later. The cement spacer is replaced by the nonvascularized bone graft. Soft tissue repair is often performed with flap coverage in the first stage. However, free flap surgeries are relatively contraindicated in patients with vascular insufficiency. As an alternative, cross-leg pedicle flap is a promising bailout for soft tissue repair. Its advantages include high flap survival rate, short operation time, and irrelevant to vessel condition in trauma limb [8]. The incidence of joint stiffness was reported less commonly in young individuals [9].

Masquelet introduced a technique to treat segmental bone loss [5]. Proponents advocated that the biological membrane induced by the cement spacers enhance thevascularity and corticalization of bone graft and facilitate bone union. The Masquelet technique offers several advantages. 1. The early application of antibiotic-impregnated cement spacers expedites local delivery of antibiotics. 2. The limb length and rotation are maintained by using internal fixation devices as early as possible. The Masquelet technique combined with flap coverage has also been studied. Li et al. retrospectively evaluated 18 cases of diaphyseal bone defect treated with bone reconstruction and flap coverage. All patients in these cases achieved good bone union [10]. Alassaf et al. reported a child with a 10-cm bone defect open fracture who was successfully treated using the Masquelet technique and a radial forearm flap coverage [3]. In our case, we performed adequate debridement in the first place. Subsequently, we restored the length and rotation of the proximal tibia through double plating and used antibiotic cement as a spacer, followed by soft tissue coverage with cross-leg pedicle flap. The cement spacer was replaced by mixed autogenous and synthetic bone graft after 12 weeks according to the principle of Masquelet technique.

The cross-leg pedicle flap is a historical flap for treating lower extremity trauma [11]. When free flap surgeries are relatively contraindicated, the cross-leg flap can be a worthy alternative to reconstruct lower extremity defects [9]. Though complications as pin tract infection, joint stiffness, deep vein thrombosis and pressure sore may occur [12], our experience showed that adequate local hygiene and vigorous ankle pumping exercise effectively prevent these complications. Our outcome demonstrates that the Masquelet technique in combination with cross-leg pedicle flap is an effective and safe method to treat Gustilo type IIIC open fracture caused by type III shotgun injury with large-scale bone and soft tissue defects.

## 4 Conclusion

This case report highlights the value of treating segmental bone loss, large soft tissue defect and vascular injury caused by shotgun injury using the Masquelet technique in combination with cross-leg pedicle flap coverage. We recommend this effective and safe method to restore bone and soft tissue defect simultaneously without the need for microsurgery while ensuring an excellent outcome.

## Conflicts of interest

All the three authors disclose any financial and personal relationships with other people or organisations that could inappropriately influence of our work.

## Sources of funding

We have no source of funding for our research.

## Ethical approval

We have the IRB approve letter attached as supplement data.

## Consent

We have consents from the patient and authors.

## Author contribution

**Chun-Yen Chen’s** work was Data collection, and original draft.

**Yung- Cheng Chiu’s** work was study design and methodology providing.

**Cheng-En Hsu’s** work was data analysis or interpretation and writing the paper.

## Research studies

Out was not a human study.

## Guarantor

Yung- Cheng Chiu AND Cheng-En Hsu.

## Provenance and peer review

Not commissioned, externally peer-reviewed.
